# Sheep Model for Uterine Transplantation: The Best Option Before Starting a Human Program

**DOI:** 10.6061/clinics/2017(03)08

**Published:** 2017-03

**Authors:** Wellington Andraus, Dani Ejzenberg, Rafael Miyashiro Nunes dos Santos, Luana Regina Baratelli Carelli Mendes, Rubens Macedo Arantes, Edmund Chada Baracat, Luiz Augusto Carneiro D’Albuquerque

**Affiliations:** I Faculdade de Medicina da Universidade de São Paulo, Departamento de Gastroenterologia, Divisão de Transplante de Orgãos Digestivos, São Paulo/SP, Brazil; II Faculdade de Medicina da Universidade de São Paulo, Departamento de Ginecologia, São Paulo/SP, Brazil

**Keywords:** Uterine transplantation, Uterus, Transplantation, Sheep, Infertility

## Abstract

**OBJECTIVE:**

This study reports the first four cases of a uterine transplant procedure conducted in sheep in Latin America. The aim of this study was to evaluate the success of uterine transplantation in sheep.

**METHOD:**

The study was conducted at Laboratory of Medical Investigation 37 (LIM 37) at the University of São Paulo School of Medicine. Four healthy mature ewes weighing 40-60 kg were used as both the donor and recipient for a transplant within the same animal (auto-transplant). Institutional guidelines for the care of experimental animals were followed.

**RESULTS:**

The first two cases of auto-transplant were performed to standardize the technique. After complete uterine mobilization and isolation of the blood supply, the unilateral vascular pedicle was sectioned and anastomosed on the external iliac vessels. After standardization, the protocol was implemented. Procurement surgery was performed without complications or bleeding. After isolation of uterine arteries and veins as well as full mobilization of the uterus, ligation of the distal portion of the internal iliac vessels was performed with subsequent division and end-to-side anastomosis of the external iliac vessels. After vaginal anastomosis, the final case presented with arterial thrombosis in the left uterine artery. The left uterine artery anastomosis was re-opened and flushed with saline solution to remove the clot from the artery lumen. Anastomosis was repeated with restoration of blood flow for a few minutes before another uterine artery thrombosis appeared on the same side. All four animals were alive after the surgical procedure and were euthanized after the experimental period.

**CONCLUSION:**

We describe the success of four uterine auto-transplants in sheep models.

## INTRODUCTION

Reproductive medicine has made extraordinary progress since the birth of the first *in vitro* fertilization (IVF) baby, Louise Brown, in 1978 1. After the introduction of intra-cytoplasmic sperm injection (ICSI) 2, most male and female infertility subtypes became treatable.

However, one subgroup of women lacked treatment options despite this progress. Women with uterine factor infertility (AUFI) have either congenital uterine absence due to Mayer-Rokitansky-Küster-Hauser (MRKH) syndrome or hysterectomy (obstetric bleeding or cervical malignancy) or a non-functional uterus caused by Asherman’s syndrome, a major malformation of the uterus or a radiotherapy injury 3. The prevalence of AUFI is approximately 3-5% in the general female population 4, and treatment of infertility in this subgroup of women is difficult 5.

The only current options for these women to become mothers are adoption or gestational surrogate carriers. Although gestational surrogacy is a method that can achieve genetic motherhood, it is not legal in some countries.

Uterus transplantation has emerged as a new type of transplant that provides an excellent option for women with lack of a functional womb and a desire to give birth 6-9. One center has already achieved great clinical success with seven transplants 10 and four live births that were successfully reported at the time of writing 9,11.

Experimental models are very important tools for further study of this transplant modality, and expertise must be acquired for use in clinical procedures 3,6-8,12. Sheep are predominantly used for this purpose due to their anatomic similarity to women.

The aim of this study was to evaluate the success of the technique for uterine auto-transplant in sheep.

## METHODS

### Animals

The study was conducted at Laboratory of Medical Investigation 37 (LIM 37) at the University of São Paulo School of Medicine and was approved by the Ethics Committee of Animal Use at the university (authorization number 063/16).

We used four healthy mature ewes weighing 40-60 kg as both donors and recipients in the same surgery (auto-transplant). Food was withdrawn 24 hours before the procedure. Institutional guidelines for the care of experimental animals were followed.

### Anesthesia

The animals underwent general anesthesia using intramuscular midazolam (0.5 mg/kg, pre-anesthetic), propofol (5 mg/kg) and isoflurane (1.5%) in 100% oxygen with a continuous infusion of fentanyl (10 µg/kg/min, iv) and a neuromuscular blockade with pancuronium (0.05 mg/kg, iv).

The ewes were subjected to mechanical ventilation with a 10 mL/kg adjustment of the respiratory rate to maintain the exhaled concentration of CO_2_ between 35 and 45 mmHg.

After orotracheal intubation, a large tube was inserted inside the esophagus to reduce the quantity of gas inside the rumen. Invasive arterial blood pressure monitoring was used during the procedure. To compensate for fluid loss during the surgical procedure, animals received a continuous infusion of Ringer Lactate solution through a central venous line.

### Surgery

#### Procurement

After general anesthesia was applied, ewes were positioned in dorsi-ventral recumbency, their abdominal wool was clipped and surgical drapes were placed. The urinary bladder was catheterized through the urethra with a Foley catheter. An inferior midline incision was used. The uterus was mobilized and exposed, and both utero-ovarian ligaments were ligated and transected.

The uterine vessels were identified and dissected bilaterally up to their origin in the iliac vessels after the mesosalpinx was transected. One difficulty was the preliminary identification of the ureter and careful bilateral separation of it from the uterine vein.

The internal iliac arteries were dissected and exposed for 5-10 mm on both sides of the insertion of the uterine artery. The medial umbilical ligament in the obliterated hypogastric artery acted as a reference for the dissection of the uterine iliac artery in the regions near the uterine artery. The external iliac vessels were dissected and exposed to facilitate the subsequent graft implantation.

After complete isolation of the vascular supply to the uterus, the internal iliac arteries were ligated proximally and distally to the uterine artery insertion to allow arterial patch creation, and a cut was made. The veins were ligated near their insertion and cut. The graft was then retrieved after retrieval of a section of the upper vagina caudal to the cervix and prepared for the implantation step during the back bench procedure. The animal was heparinized prior to uterus retrieval.

#### Bench surgery

**Figure 1:**
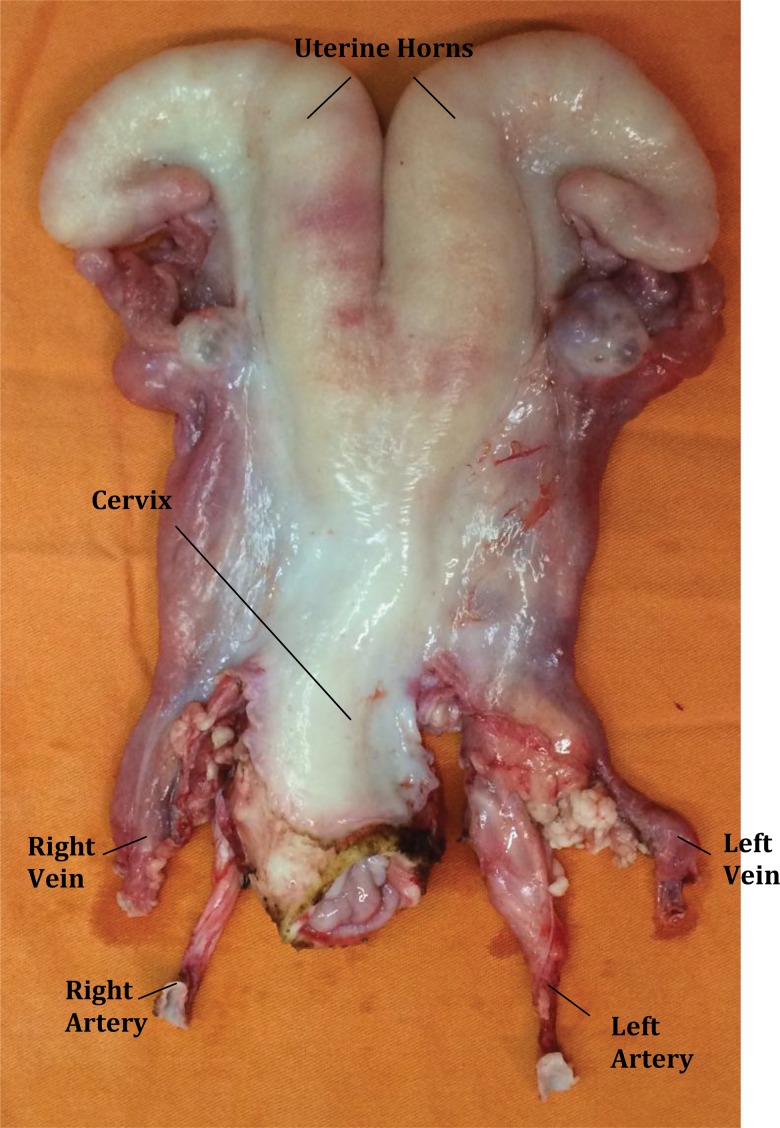
Bench surgery of the ovine uterus for auto-transplantation.

The distal portions of the internal iliac artery were cut for creation of an arterial patch on both sides. The uterine artery was catheterized and subsequently perfused on both sides with Ringer Lactate solution until complete discoloration of the uterus was achieved (Figure 1). Heparin was also added to the Ringer’s solution (5000 U/L) to minimize thrombosis.

#### Implantation

The small bowel, colon and rumen were retracted to the upper abdomen using large gauze. Vascular clamps were placed in the external iliac vessels on one side at a time. The anastomosis was started in the uterine vein and proceeded to the uterine artery.

The vessels were opened with a scalpel and washed with saline solution before anastomosis was performed. Continuous 7-0 polypropylene end-to-side anastomoses were performed for both the vein and artery. After completion of the first side, the other side was submitted to the same procedure; the clamps were released after conclusion of all vascular anastomoses, and hemostasis was evaluated. Anastomotic patency and blood flow were assessed by visual inspection of uterine color and pulsatile evaluation of the uterine arteries. Anastomosis of the vagina was performed with a 3-0 vicryl continuous suture. The uterus was then fixed to the peritoneum and ligaments.

After the end of the implantation, all anastomosis and blood flow parameters were reassessed, and the animals were euthanized by increasing the isoflurane concentration from 1.5% to 5% and through KCl intravenous administration.

## RESULTS

**Figure 2:**
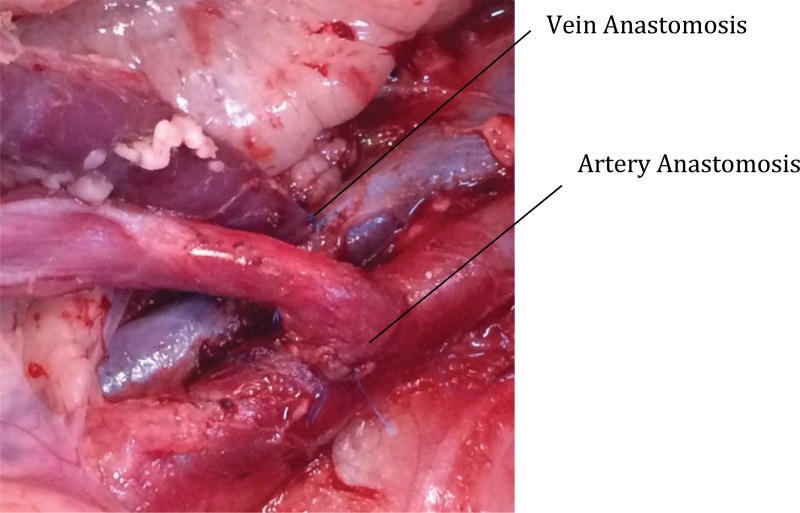
Anastomosis of uterine vessels in the external iliac vessels.

Four auto-transplant procedures were performed. Procurement surgery was performed without complications in all four cases. The first two cases were performed in February 2016 for standardization of the technique. After complete uterine mobilization and isolation of the blood supply, the unilateral vascular pedicle was sectioned and anastomosed on the external iliac vessels (Figure 2).

**Figure 3:**
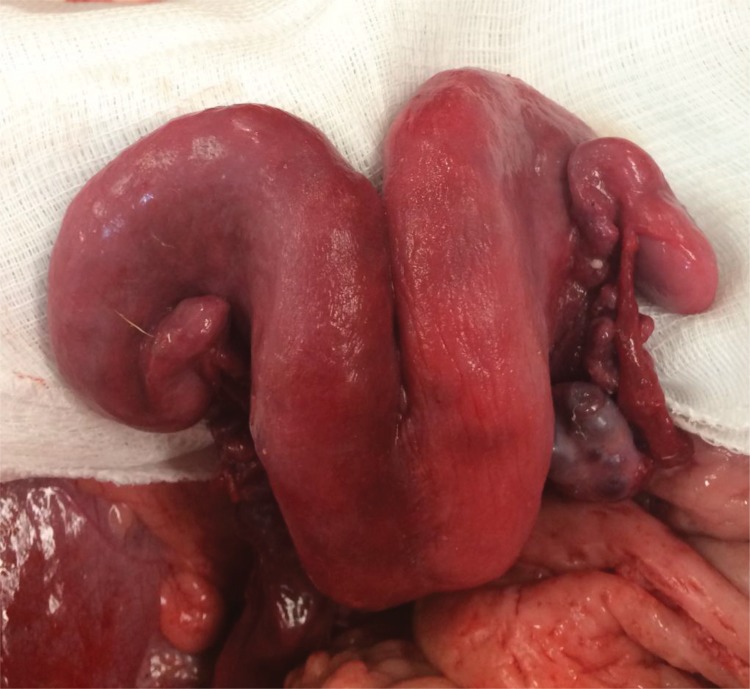
Image of the uterus after reperfusion.

No complications were reported in these procedures. Complete restoration of blood flow was achieved after end-to-side anastomosis while maintaining good pulsation on the anastomosed uterine artery. The uterine aspect was bilaterally similar, which confirmed restoration of blood flow after successful vascular anastomosis (Figure 3).

After the standardization procedures, the protocol was fully implemented. Procurement surgery was performed as described in the methods section without complications or bleeding. After isolation of uterine arteries and veins and full mobilization of the uterus, ligations of distal portions of the internal iliac vessels were performed with their subsequent division. The vagina was sectioned close to the cervix, and the graft was transported to the bench for flushing.

Minimal bleeding occurred during the entire procedure. We used monopolar and bipolar cautery to perform careful hemostasis. During the isolation of the uterus from the bladder, the bladder became dark, but normal blood supply was maintained to the uterus.

The bench procedure was easily and quickly performed. During our first attempt, we perfused the uterus with gravity, but the perfusion was slow and inefficient. After modifying the protocol to perfuse the uterus manually using a 20 cc syringe, much better results were achieved with fast blanching of the uterus color (Figure 1).

The implantation surgery was performed according to the methods section of this paper. The mean anastomosis time was 13 min per vessel. Minor bleeding from the arterial suture was present in one case, which was easily corrected by one extra separated 7-0 Prolene stitch.

After vaginal anastomosis, one case presented an arterial thrombosis in the left uterine artery. The pulsation in the artery stopped, and the color of the left uterine horn became darker. The left uterine artery anastomosis was re-opened, and the artery lumen was flushed with saline solution containing clot-removing agents. Anastomosis was repeated with the restoration of blood flow for a few minutes before a new uterine artery thrombosis occurred on the same side. After this case, we increased the dose of the heparin infusion before retrieval and included heparin in the Ringer’s solution.

The procedure was considered successful in all animals at the end of the experiment.

## DISCUSSION

To the best of our knowledge, this is the first report of a womb transplant performed in sheep in Latin America. The sheep model was chosen due to its similarities to human anatomy. Non-human primates were not considered for our model because they are prohibitively expensive and more prone to zoonosis, and restrictions on the use of these animals in research exist. Our model was similar to the model described by Saso et al., which was designed in preparation for clinical trials in the United Kingdom 13, in which 5 sheep auto-transplant attempts were made, and 3 animals were successfully reperfused.

We performed a bilateral anastomosis of the uterine arteries and veins (4 anastomoses) to maintain the original length and directions of the vessels and reduce the risk for thrombosis. Aortal and caval patches could reduce the number of anastomoses (2 anastomoses) and facilitate the procedure due to the larger size of the anastomoses, but the redundancy of the vessels can lead to the formation of torsion or kinks, which could lead to thrombosis, as previously reported 14.

Brännström et al. 15 reported a series of nine cases of living donor uterus transplantation after over a decade of experimental work with small and large animals with good success rates. His experimental models included mice, rats, pigs, sheep and non-human primates. The first model in mice resulted in pregnancy 16 and offspring 17 after syngeneic uterine transplant. Following this success, the first pregnancies and offspring were demonstrated in rat allotransplant models 18,19. Once the feasibility of uterine transplantation with immunosuppression was shown in small animals, attempts from the same research group were made in larger animals, beginning with pigs. However, their attempts were unsuccessful 20. In fact, this group also presented a high rate of vascular thrombosis in pig models 21, and thrombosis was only partially solved by utilizing a macrovascular patch that included the aorta and vena cava in the graft for anastomosis in a heterotopic uterine transplant model 22. Sheep were the next animal used as uterine transplant models. This animal has advantages over the swine model due to its similarities to the anatomy and size of the uterine vasculature in humans 23. The Brännström group developed an auto-transplant model with a single uterine horn vascularized pedicle anastomosed in the ipsilateral external iliac vessels 24, and they achieved live births 25 using this technique.

Once offspring are produced following transplantation in large animals, the last step before clinical uterine transplantation is the development of a non-human primate model to determine the best flushing technique 26,27 and immunosuppression protocols 28. Our group is preparing to initiate a clinical uterine transplantation program, and similar to the Brännström group, we are developing the technique using animal models before clinical attempts. In this manuscript, we described a modified sheep model that was consistent with a successful human model using uterine arteries and veins and performing vascular anastomosis in recipient external iliac vessels (auto-transplant).

Our study provides the first description of this technique in Latin America using a model that could be reproduced in other centers, and practice with this model should be part of a human transplantation program. This study had some limitations, such as the low number of animals that were used, the inability to evaluate survival rates due to animal euthanasia, and no functional study was conducted for the transplanted uterus.

We described the success of four uterine auto-transplants in sheep models. Further studies are necessary to evaluate the functions of this uterine auto-transplant method.

## AUTHOR CONTRIBUTIONS

Andraus W, Ejzenberg D, dos Santos RM, Mendes LR and Arantes RM conceived and designed the study. Baracat EC and D’Albuquerque LA reviewed the manuscript. Andraus W, Ejzenberg D and dos Santos RM were responsible for the data analysis and interpretation. Andraus W was responsible for the final approval of the manuscript.
